# Antimicrobial resistance, virulence gene, and biofilm formation of *Escherichia coli* isolated from yaks in Qinghai, China

**DOI:** 10.3389/fvets.2026.1894554

**Published:** 2026-10-02

**Authors:** Shengyi Han, Qiong Li, Tian Shi, Wenwen Zhou, Gangzhu Jin, Ziwei Hu, Shinang Zhang, Kailun Zhang, Rongfei Yang, Guoyuan Hu, Chunyan Xu, Shengqing Li

**Affiliations:** 1 Qinghai Academy of Animal Sciences and Veterinary Medicine, Qinghai University, Xining, China; 2 Qinghai Provincial Key Laboratory of Pathogen Diagnosis for Animal Disease and Green Technical Research for Prevention and Control, Xining, China; 3 College of Veterinary Medicine, Henan Agricultural University, Zhengzhou, China; 4 Ministry of Education Key Laboratory for Animal Pathogens and Biosafety, Zhengzhou, China; 5 Key Laboratory of Quality and Safety Control of Poultry Products, Ministry of Agriculture and Rural Affairs, Zhengzhou, China

**Keywords:** antimicrobial resistance, biofilm formation, phylogenetic analysis, virulence genes, yak-derived *Escherichia coli*

## Abstract

**Introduction:**

This study investigated the antimicrobial resistance (AMR), virulence gene profiles, and biofilm formation capabilities of *Escherichia coli* (*E. coli*) isolated from yaks in Qinghai, China.

**Methods:**

A total of 60 isolates were analyzed for resistance against 11 antibiotics. PCR was used to identify antibiotic resistance genes (ARGs) and virulence genes. Biofilm formation was assessed via phenotypic assays. Phylogenetic typing and serotyping were performed using standard molecular and immunological methods.

**Results:**

High prevalence of multidrug resistance (MDR) was observed, particularly to tetracyclines (43.33%), ciprofloxacin (33.33%), and cefotaxime (33.33%). A total of 63 ARGs were identified, with *bla*_EC-18_ (85%) being the most prevalent. Virulence gene screening detected 253 factors, including *csgA*, *csgC*, and *entD* (100% prevalence). Biofilm assays showed that 40% of isolates were strong biofilm producers. Phylogenetic typing classified most strains as phylogroup B1 (81.67%), with sequence types ST2522 (8.33%) and ST56/ST10 (5% each) being dominant. Serotyping revealed O25 and O107 as the most common serotypes (16.67% each). Strong biofilm formers exhibited higher ARG diversity, including *bla*_EC-18_ (79.17%) and *sul*1 (41.67%).

**Conclusion:**

Overall, these findings identify diarrheic yak‑associated *E. coli* as a potential reservoir of resistance and virulence determinants, and support strengthened antimicrobial stewardship, farm hygiene, and One Health surveillance in the Qinghai‑Tibet Plateau.

## 1 Introduction

The yak (*Bos grunniens*) is the most important livestock species in the high-altitude ecosystems of the Himalayas and Qinghai-Tibet Plateau, where it provides meat, milk, hides, wool, and transportation for local herders (1–4). China holds approximately 95% of the global yak population, and Qinghai Province alone had 5.8 million yaks by the end of 2022, accounting for 35% of the national total and earning the region recognition as the “Global Yak Capital” (5, 6). However, the harsh plateau environment, seasonal nutritional stress, extensive grazing, and limitations in veterinary services can increase the susceptibility of young and adult yaks to enteric disease. Diarrhea is a common clinical problem in yak production and can cause growth retardation, treatment costs, and mortality, thereby reducing the sustainability of plateau animal husbandry. Among the bacterial agents associated with enteric disease, *E. coli* is of particular concern because it includes both commensal lineages and pathogenic variants capable of intestinal colonization, epithelial injury, toxin production, and systemic dissemination.

Yak Colibacillosis is an acute intestinal infectious disease caused by pathogenic *E. coli*, primarily characterized by severe diarrhea, dehydration, and acute septicemia (7, 8). As a gram-negative bacterium of the Enterobacteriaceae family, *E. coli* is a conditional pathogen widely distributed in the intestinal tracts of all vertebrates (9). Ruminants serve as one of the most significant reservoirs for pathogenic *E. coli*, with transmission typically occurring through cross-contamination of water, feed, and feces, affecting both animals and humans (10). Diarrhea represents a prevalent gastrointestinal disorder in yak farming, with pathogenic *E. coli* being the predominant etiological agent (11). In recent years, *E. coli* has emerged as a common opportunistic pathogen in human and animal intestines due to its extensive antibiotic resistance mechanisms and numerous serotypes (12, 13). Furthermore, its susceptibility to environmental factors renders free-ranging yaks on the plateau particularly vulnerable to colibacillosis, thereby indirectly impacting herders’ livelihoods and health.

*Escherichia coli* strains are conventionally classified into phylogenetic groups A, B1, B2, and D. However, recent phylogenetic analyses have validated the division of *E. coli* strains into eight phylogenetic groups: four primary groups (phylotypes A, B1, B2, and D) and four newly identified groups (C, E, F, and clade I) (14, 15). Groups B2 and D are associated with extraintestinal infections and are considered more invasive strains. Consequently, phylogenetic classification of *E. coli* strains is valuable not only for characterizing *E. coli* populations but also for elucidating the correlation between phylotypes and disease manifestations caused by the pathogen. The pathogenicity of *E. coli* stems from its diverse virulence factors (VFs), including adhesins, host cell surface modifiers, invasins, toxins, and secretion systems. These virulence factors can undergo horizontal gene transfer via plasmids, bacteriophages, and transposons, conferring broad pathogenic potential (16, 17). In addition to VFs, antibiotic resistance and biofilm formation further complicate the control of *E. coli* infections (18).

Antibiotics represent the most potent tools for controlling and preventing *E. coli* infections and contamination. However, the widespread use of antibiotics has led to the global emergence of resistant and multidrug-resistant *E. coli* strains, posing significant threats to public health and safety. Biofilm formation disrupts host cellular immune mechanisms by enhancing bacterial resistance to host immune defenses and antibiotic tolerance (19, 20). This process reduces the exposure of pathogen-associated molecular patterns (PAMPs), enabling immune evasion by circumventing host receptor recognition (21). Furthermore, biofilms serve as critical virulence determinants in *E. coli*. Key biofilm-associated genes-including *crl*, *csgA*, *fimH*, *luxS*, and *papC*-function as essential virulence factors that facilitate host adhesion and infection initiation. These genes promote bacterial colonization, invasion, and community formation, thereby enhancing pathogenicity and frequently contributing to chronic infections (22).

The O-antigen, a major virulence factor influencing bacterial survival, invasiveness, and pathogenicity, exhibits serotype-specific associations with disease potential. To date, over 180 O-serotypes of *E. coli* have been identified based on the highly variable structure of O-antigens. Notably, loss of O-antigen renders many pathogens serum-sensitive or severely attenuates their virulence (23). Consequently, understanding the distribution of *E. coli* O-antigen serotypes is critical for effective disease prevention and control.

Despite the economic and ecological importance of yaks, the molecular epidemiology of *E. coli* associated with yak diarrhea remains insufficiently characterized. Existing studies in China have often focused on antimicrobial susceptibility or a limited panel of resistance and virulence genes, whereas systematic information on genomic resistance determinants, plasmid replicons, serotypes, phylogenetic groups, STs, and their relationships with biofilm-forming capacity remains scarce. In particular, it is unclear whether diarrheic yaks in Qinghai harbor globally distributed lineages, locally enriched clones, or strains combining persistent biofilm phenotypes with clinically relevant resistance and virulence determinants. This knowledge gap restricts evidence-based treatment, epidemiological risk assessment, and the development of region-specific surveillance and control strategies.

Accordingly, the present study investigated 60 *E. coli* isolates recovered from diarrheic yaks in Qinghai, China. We characterized their antimicrobial susceptibility profiles, ARGs, virulence-factor repertoires, plasmid replicons, phylogroups, STs, serotypes, and biofilm-forming capacities, and further compared the distributions of resistance and virulence determinants among isolates with different biofilm phenotypes. By integrating phenotypic and genomic data, this study aimed to define the major resistance patterns and population structure of yak-derived *E. coli*, identify potentially persistent or epidemiologically important lineages, and provide baseline evidence for rational antimicrobial use, farm-level biosecurity, and One Health surveillance in the Qinghai-Tibet Plateau.

## 2 Materials and methods

### 2.1 Isolation and identification of *Escherichia coli*

A total of 99 samples were collected from diarrheic yaks across six regions of Qinghai, China, namely Qumalai, Yushu, Zhongduo, Haiyan (Haibei Prefecture), Maqên, and Maqên County Pasture. The sampled animals ranged in age from less than 1 week to 1 year. All yaks were raised under traditional grazing systems, with each pastoral farm maintaining a herd size of approximately 100–300 animals. In these regions, the antimicrobials most commonly used in yak husbandry include oxytetracycline, cefotaxime, and enrofloxacin. The collected specimens comprised anal swabs from calves with active diarrhoea and intestinal contents from deceased calves. From these samples, 60 strains of *E. coli* were isolated through a combination of Eosin Methylene Blue (EMB) and SS media, Gram staining, biochemical identification, and 16S rDNA sequencing.

### 2.2 Antimicrobial susceptibility testing

Antimicrobial susceptibility testing was performed on the isolated yak-derived *E. coli* strains using the Kirby-Bauer disk diffusion method according to Clinical and Laboratory Standards Institute (CLSI) guidelines. The antibiotic panel included 11 agents representing six major classes: Tetracyclines: Tetracycline (TCY, 30 μg); Fluoroquinolones: Ciprofloxacin (CIP, 5 μg), Ofloxacin (OFX, 10 μg), Norfloxacin (NOR, 10 μg); Aminoglycosides: Kanamycin (KAN, 30 μg), Amikacin (AMK, 30 μg), Gentamicin (GEN, 10 μg); *β*-lactams: Ceftriaxone (CRO, 30 μg), Ampicillin (AMP, 10 μg); Cephalosporins: Cefotaxime (CTX, 30 μg); Sulfonamides: Trimethoprim-sulfamethoxazole (SXT, 1.25/23.75 μg). All antimicrobial disks were obtained from Hangzhou Binhe Microbial Reagent Co., Ltd. (Hangzhou, China). *E. coli* ATCC 25922 (purchased from the China Institute of Veterinary Drug Control, Beijing) served as the quality control strain. Results were interpreted according to CLSI M100 (2022) breakpoints (Table 1). Multidrug resistance (MDR) was defined as acquired resistance to ≥1 agent in ≥3 antimicrobial categories.

**Table 1:** Criterion of antibiotics sensitivity test.

Antimicrobial agent	Disk content	Interpretive categories and zone diameter breakpoints nearest whole mm		
		S	I	R
Tetracycline	30 μg	≥15	12–14	≤11
Ciprofloxacin	5 μg	≥31	21–30^	≤20
Ofloxacin	10 μg	≥16	13–15^	≤12
Kanamycin	30 μg	≥18	14–17^	≤13
Ceftriaxone	30 μg	≥23	20–22^	≤19
Cefotaxime	30 μg	≥26	23–25^	≤22
Amikacin	30 μg	≥17	15–16^	≤14
Gentamicin	10 μg	≥15	13–14^	≤12
Ampicillin	10 μg	≥17	14–16^	≤13
Norfloxacin	10 μg	≥17	13–16	≤12
Trimethoprim-sulfamethoxazole	1.25/23.75 μg	≥16	11–15	≤10

### 2.3 Serotyping of O antigens in isolated strains

Antigen preparation: Purified *E. coli* isolates were cultured on LB agar at 37 °C for 24 h. Bacterial lawns were harvested using 2 mL of 0.5% phenol-saline solution, and the resulting suspensions were autoclaved at 121 °C for 2 h to eliminate K antigen interference, thereby preparing the test antigens.

Serological testing: O-antigen serotyping was performed using 59 monovalent O-antigen antisera (O1, O2, O4, O5, O6, O8, O9, O10, O11, O15, O20, O21, O22, O24, O25, O26, O27, O30, O33, O35, O36, O44, O45, O55, O64, O77, O78, O86, O88, O91, O104, O107, O111, O115, O116, O117, O118, O125, O126, O127, O135, O138, O142, O143, O144, O145, O148, O149, O152, O153, O154, O157: H7, and O158) purchased from the China Institute of Veterinary Drug Control, stored at 2–8 °C and used within the validity period. Heat-treated antigens were tested by slide agglutination with these antisera: 10 μL of antiserum was mixed with a fresh single colony on a glass slide and observed visually within 1 min, with physiological saline as the negative control. A positive reaction (+) was defined as distinct granular agglutination with the antiserum while the saline control remained uniformly turbid; a negative reaction (−) was defined as uniform turbidity without visible agglutination particles. Strains that also agglutinated in the saline control were considered auto-agglutinable and excluded. All reactions were performed in duplicate at room temperature (20–25 °C) to ensure consistent results.

### 2.4 Phylogenetic typing

Phylogenetic classification of *E. coli* isolates was performed using multiplex PCR targeting chuA, yjaA, and TspE4.C2 genes according to the Clermont typing scheme. The primer sequences for these genetic markers are listed in Table 2. All oligonucleotide primers were commercially synthesized by Tsingke Biotechnology Co., Ltd. (Xi’an, China). The PCR amplification was carried out in a 25 μL reaction mixture containing: 1 μL of each primer pair (forward and reverse), 3 μL of bacterial DNA template, 12.5 μL of 2 × Taq Master Mix, and 3.5 μL of ddH₂O. The thermal cycling conditions consisted of an initial denaturation at 94 °C for 5 min; followed by 30 cycles of denaturation (94 °C, 30 s), annealing (30 s), and extension (72 °C, 30 s); with a final extension at 72 °C for 7 min. PCR products were electrophoresed on 1.5% agarose gels for visualization. The phylogenetic groups (A, B1, B2, and D) were determined based on specific combinations of chuA, yjaA, and TspE4.C2 amplification patterns, as detailed in Table 3.

**Table 2:** Primer information for phylogenetic grouping analysis.

Primer	Sequence (5′ → 3′)	Length (bp)	Annealing temperature (°C)
*ChuA*	GACGAACCAACGGTCAGGAT	279	55
	TGCCGCCAGTACCAAAGACA		
*YjaA*	TGAAGTGTCAGGAGACGCTG	211	55
	ATGGAGAATGCGTTCCTCAC		
*TspE4.C2*	GAGTAATGTCGGGGCATTCA	152	55
	CGCGCCAACAAAGTATTACG		

**Table 3:** Classification scheme for *Escherichia coli* phylogenetic groups.

Phylogenetic groups	*ChuA*	*YjaA*	*TspE4.C2*
A	**−**	**−/+**	**−**
B1	−	**−/+**	**−**
B2	**+**	**+**	**−/+**
D	**+**	**−**	**−/+**

### 2.5 Biofilm formation ability assay

Based on previous studies, the crystal violet microtiter plate method was employed for the detection and quantification of biofilm (BF) formation ability (24–26). Overnight cultures were diluted at a ratio of 1:100 in fresh LB broth, and 300 μL aliquots were transferred to a 96-well plate. The plate was then incubated at 37 °C with shaking at 150 r/min for 12 h. Each bacterial strain was tested in triplicate wells, with *E. coli* K-12 MG1655 as the negative control and *E. coli* ATCC 35218 as the positive control. Following incubation, the LB broth was discarded, and the wells were washed three times with sterile PBS buffer and allowed to air-dry. Biofilms were fixed with 100% methanol for 5 min. After decanting the methanol, the wells were air-dried again and stained with 0.1% (w/v) crystal violet for 15 min. Residual crystal violet was gently rinsed off with distilled water, and the wells were air-dried. The biofilms were then solubilized with 33% glacial acetic acid, and the absorbance at 595 nm (OD595 nm) was measured. Each measurement was repeated three times. The cutoff OD value (ODc) for determining biofilm formation was calculated as the mean absorbance of the negative control plus three times the standard deviation. The biofilm formation ability of the test strains was assessed by comparing their OD595 nm absorbance values to the ODc value. The criteria for classification are presented in Table 4. The biofilm formation ability of the isolated and identified *E. coli* strains was evaluated. Additionally, we performed Spearman’s rank correlation analysis because biofilm-forming capacity was recorded as an ordinal variable. ARG diversity was defined as the number of distinct ARGs detected per isolate, and the virulence gene carriage rate was calculated as the proportion of the examined virulence genes detected in each isolate.

**Table 4:** Presents the criteria for the classification of biofilm formation ability in bacterial strains.

Biofilm formation capacity	Judging criteria
None (−)	OD595nm<ODc
Weak (1+)	ODc<OD595nm<2 ODc
Moderate (2+)	2 ODc<OD595nm<4 ODc
Strong (3+)	OD595nm>4 ODc

### 2.6 Whole genome sequencing and data analysis

#### 2.6.1 Whole genome sequencing

The isolated *E. coli* strains from yaks were subjected to library construction and sequencing using the Illumina MiSeq platform (completed by Shanghai Personal Biotechnology Co., Ltd.). Data quality control was performed using FastQC. Sequence assembly of high-quality sequencing raw data was conducted using the Spades software. Prediction of open reading frames (ORFs) and their annotations was performed using Prokka v1.14.6 (27, 28).

#### 2.6.2 Sequence type (ST) analysis

Housekeeping gene detection and determination of ST types for all assembled whole genome sequences were performed using MLST 2.0 on the Center for Genomic Epidemiology (CGE) platform.^1^ A minimum spanning tree (MST) of MLST data from different isolates was constructed using BioNumerics v.8.0 software (Applied Maths, Sint-Martens-Latem, Belgium).

#### 2.6.3 Detection of antimicrobial resistance genes, virulence genes, and plasmid replicons

Comparison and statistical analysis of antimicrobial resistance genes, virulence genes, and plasmid replicons in the strains were performed using the Abricate software. The NCBI antimicrobial resistance gene reference database was used for antimicrobial resistance gene detection, the Virulence Factor Database (VFDB) was used for virulence gene detection, and the Plasmidfinder database was used for plasmid replicon detection.

#### 2.6.4 Construction of single nucleotide polymorphism (SNP) phylogenetic trees and visualization of gene distribution Heatmaps

SNP-based phylogenetic trees were constructed using Parsnp software based on single nucleotide polymorphism differences in the core genomes of the strains. The phylogenetic trees were visualized using the website https://www.chiplot.online/. Gene distribution heatmaps were generated using TBtools-II.

## 3 Results and discussion

### 3.1 Phenotypic antibiotic resistance

The 60 *E. coli* isolates from yaks exhibited varying resistance levels to 11 antibiotics (Figure 1). Among six antibiotic classes, the isolates showed high sensitivity to fluoroquinolones (ofloxacin and norfloxacin), while demonstrating >65% sensitivity to *β*-lactams (ceftriaxone), aminoglycosides (amikacin and gentamicin), and sulfonamides (trimethoprim-sulfamethoxazole). The highest resistance rate was observed for tetracyclines (43.33%), followed by ciprofloxacin (33.33%) and cefotaxime (33.33%). The high tetracycline resistance in diarrheic yak-derived *E. coli* isolates was consistent with previous findings in *E. coli* isolates from diarrheic yaks in Tibet and Qinghai, China (10, 29), but lower than that in *E. coli* isolated from duck farm soils in China (30). The resistance rates to trimethoprim-sulfamethoxazole, amikacin, gentamicin, and cefotaxime were significantly lower than those in *E. coli* isolates from healthy yak feces in Sichuan, China (9), while tetracycline and ciprofloxacin resistance rates were notably higher. Compared with *E. coli* isolates from both healthy and diarrheic yak fecal samples (31), our isolates showed higher resistance rates to tetracycline (<40% vs. 43.33%), gentamicin (<20% vs. 30.33%), and ciprofloxacin (<10% vs. 33.33%) (16). Relative to *E. coli* strains from healthy waterfowl in Hainan, China (32), our isolates exhibited lower resistance to cefotaxime and ceftriaxone, higher resistance to gentamicin, and similar resistance to ciprofloxacin (33). The resistance patterns to gentamicin and tetracycline were comparable to those in *E. coli* from diarrheic sheep (34), while tetracycline resistance was relatively lower than in *E. coli* from diarrheic dogs (35), with similar resistance rates observed for gentamicin, ciprofloxacin, and cefotaxime. Resistance rates differed significantly among the five antimicrobial classes (Cochran’s Q = 13.647, df = 4, *p* = 0.0085). The highest resistance rate was observed for *β*-lactams (46.7%, 28/60), followed by tetracyclines (43.3%, 26/60), fluoroquinolones (36.7%, 22/60), aminoglycosides (28.3%, 17/60), and folate pathway inhibitors (23.3%, 14/60). Pairwise comparisons with Holm correction showed that resistance rates to *β*-lactams and tetracyclines were significantly higher than that to folate pathway inhibitors (adjusted *p* = 0.0258 and 0.0376, respectively).

**Figure 1:**
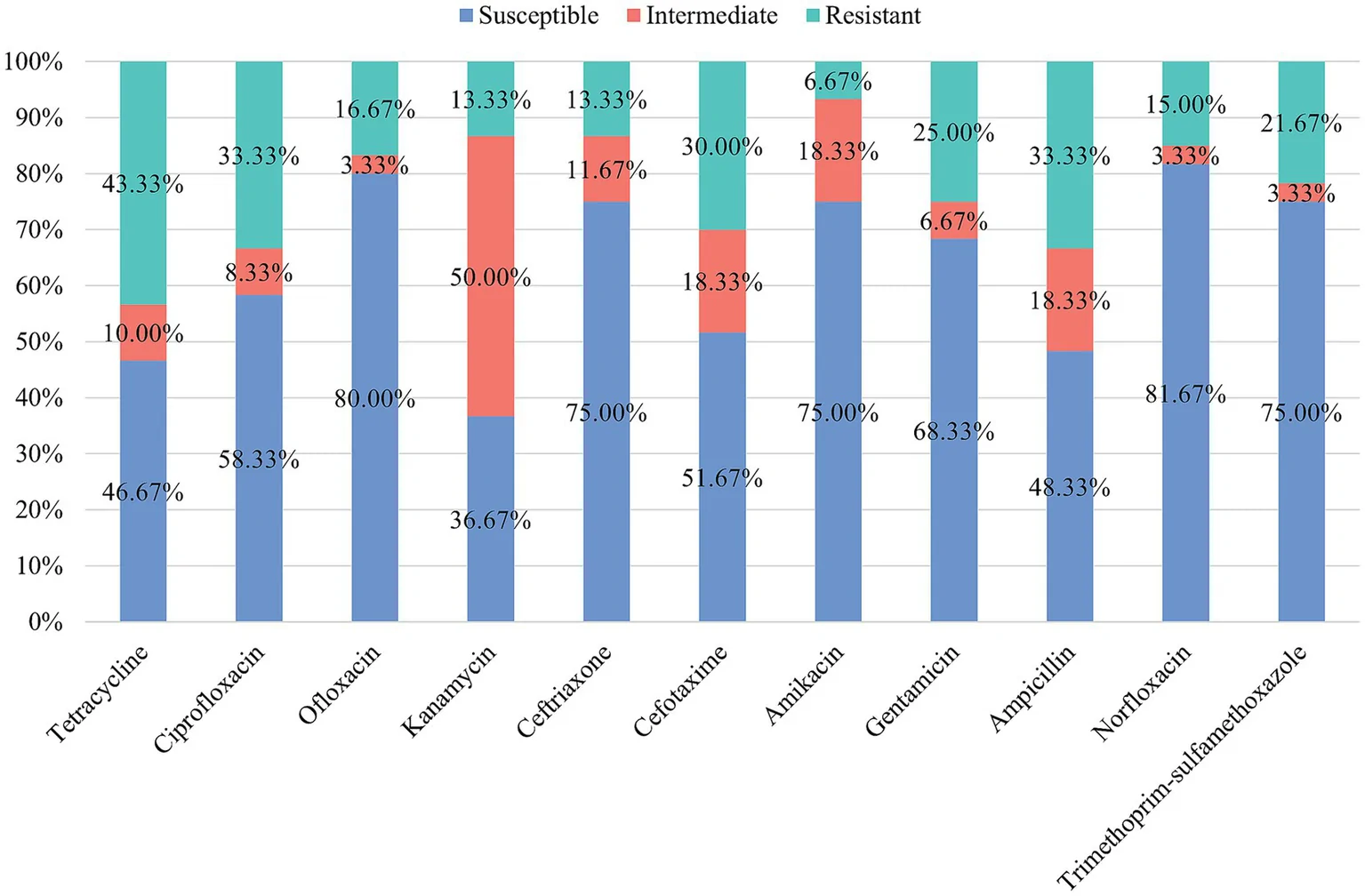
Drug resistance rate of 60 *E. coli* strains to 11 antimicrobial agents. Distribution of susceptibility phenotypes (Susceptible, Intermediate, Resistant) across 11 antibiotics. The height of each stacked segment represents the proportion (%) of isolates within that category. The susceptibility, intermediate, and resistance rates are represented by blue, red, and green, respectively.

In summary, the frequent use of tetracyclines in diarrheic animals and similar antibiotic classes in yaks may explain these resistance patterns. Consistent with a recent study on dairy farm waste in Gansu Province, which also highlighted the prevalence of aminoglycoside- and *β*-lactam-resistant *E. coli* in farm environments (34), regional variations likely contribute to the differences in certain antibiotic resistance phenotypes. However, the high resistance rates to tetracyclines, ciprofloxacin, and cefotaxime reflect the extensive and heavy use of these antibiotics in yaks.

### 3.2 Antibiotic resistance genes

The detection of resistance genes revealed that the isolates carried 12 classes of antibiotic resistance genes, comprising 63 distinct ARGs (Figure 2). Among these, the *β*-lactam resistance gene *bla*_EC-18_ showed the highest prevalence with a detection rate of 85% (51/60), followed by *bla*_TEM-1_ at 25% (15/60). For aminoglycoside resistance genes, *aph*(6)-*Id* was detected in 33.33% (20/60) of isolates, while *aph*(3″)-*Ib* was found in 31.67% (19/60). Among sulfonamide resistance genes, *sul2* and *sul1* were detected at rates of 31.67% (19/60) and 23.33% (14/60), respectively. For tetracycline resistance genes, *tet*(B) and *tet*(A) were identified in 28.33% (17/60) and 23.33% (14/60) of isolates. Resistance genes for chloramphenicol, lincosamides, glycopeptides, streptomycin, and fosfomycin all showed detection rates below 10%. The ARG profile was consistent with the observed antibiotic resistance phenotypes. In this study, the detection rate of *sul*1 (23.33%) was lower than that reported for yak-derived isolates from Sichuan Province (46%), while *sul2* detection (31.67%) was higher than previous reports (7%) (9). The *β*-lactam resistance genes showed both higher detection rates and greater diversity compared to earlier studies (31). Notably, *bla*_EC-18_ was detected in 85% of the isolates. However, *bla*_EC_ genes generally represent chromosomally encoded AmpC-family *β*-lactamase variants in *E. coli*, and the distribution of individual *bla*_EC_ alleles may be associated with the phylogenetic background of the isolates. Because phylogroup B1 predominated in the present collection, the high prevalence of *bla*_EC-18_ may partly reflect the population structure of the isolates rather than antimicrobial selection alone. Therefore, the presence of *bla*_EC-18_ should not be interpreted as direct evidence of excessive local *β*-lactam use without additional antimicrobial-use data, gene-expression analysis, or characterization of its genomic context (35). Nevertheless, the observed cefotaxime resistance and the presence of acquired *β*-lactam resistance genes such as *bla*_TEM-1_ support restricting the empirical use of extended-spectrum cephalosporins and strengthening susceptibility-guided treatment.

**Figure 2:**
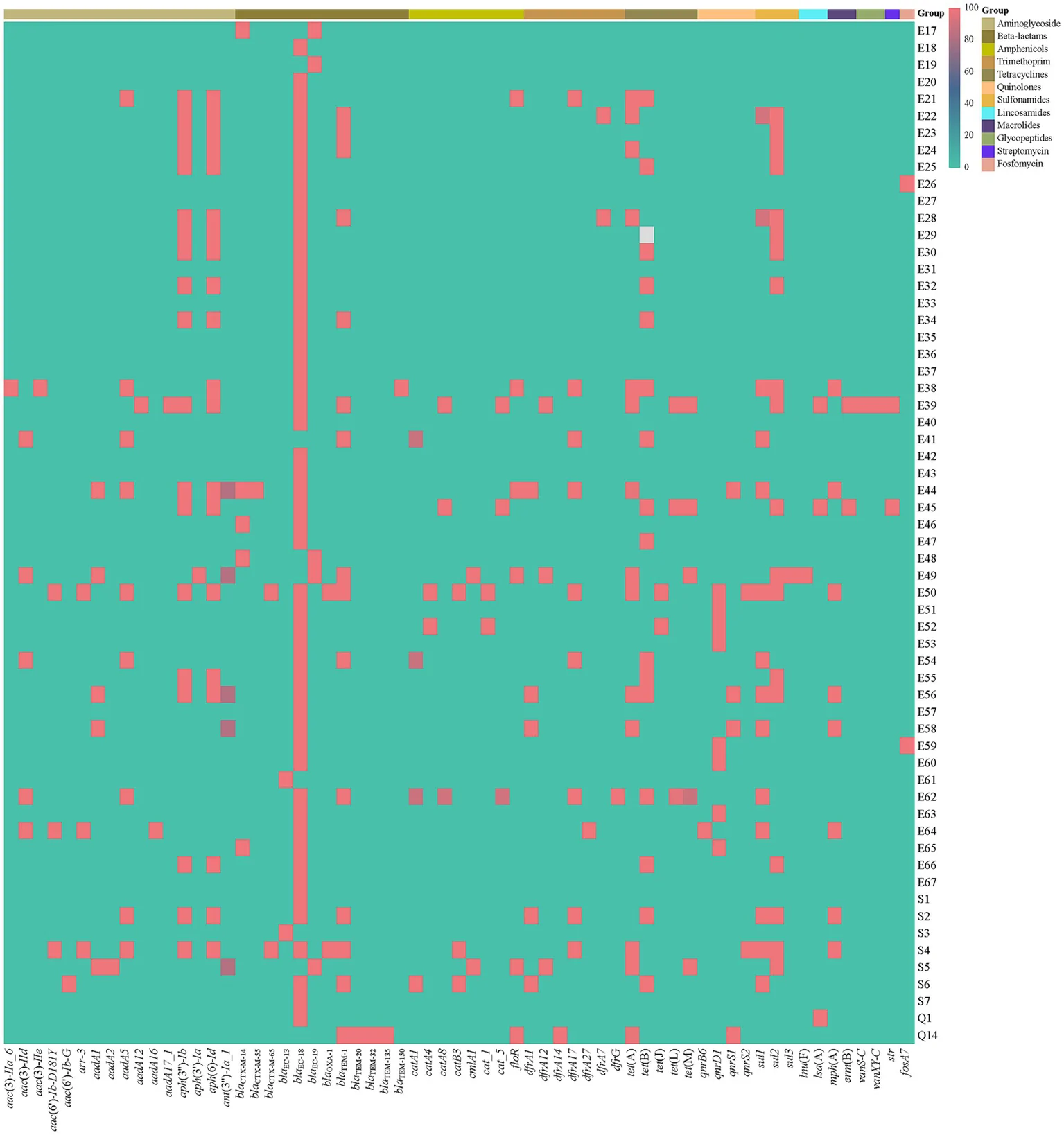
Heat map of antibiotic resistance gene analysis. The heatmap displays the presence (red) or absence (green) of resistance genes across all isolates. Colored bars above the heatmap indicate the antibiotic class to which each gene confers resistance (see color key at the top). Columns represent individual resistance genes, and rows represent bacterial isolates.

### 3.3 Virulence gene detection

The *E. coli* isolates carried 253 virulence factors (Figure 3). Among these, the genes *cgsG*, *csgA*, *csgC*, *entD*, *fdeC*, *gndA*, *phoP*, and *rpoS* were detected in all isolates (100%, 60/60). Additionally, *acrB*, *cgsD*, *entB*, *espX4*, *fepB*, *fepC*, *fepG*, *fimA*, f*imE*, *fimF*, *fimI*, *fur*, *ompA*, *pmrA*, and *yagV*/*ecpE* showed a 98.33% detection rate (59/60). Among the detected virulence factors, those associated with hemolysin (*hlyA*, *hlyB*, *hlyC*, *hlyD*), Shiga toxin production (*stx*2A, *stx*2B), the yersiniabactin pathogenicity island (*irP1*, *irP2*), the locus of enterocyte effacement (LEE) island (*eae*), and temperature-sensitive hemagglutinin (*tsh*) were all detected at rates below 35%.

**Figure 3:**
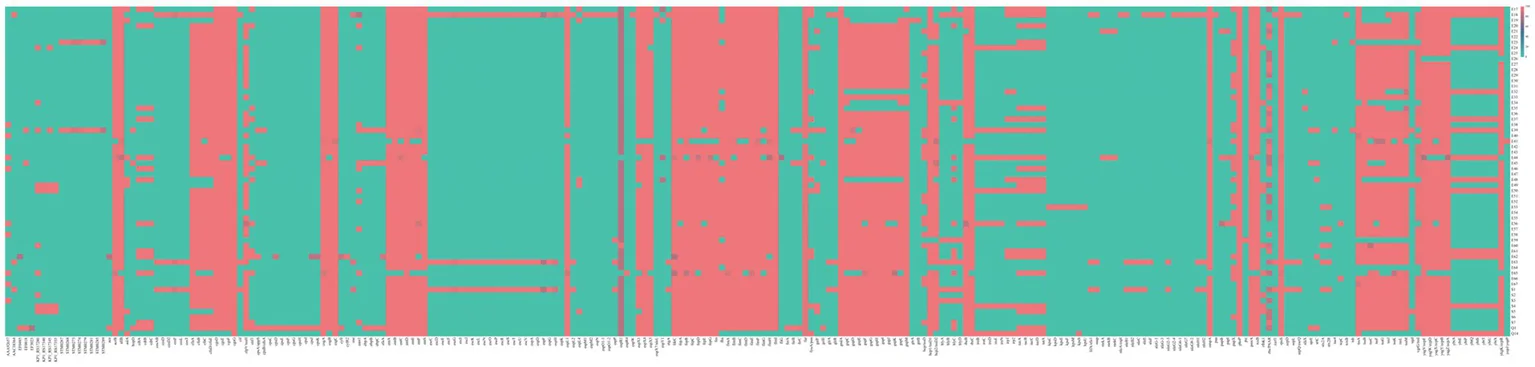
Heat map of virulence factors analysis. A heat map depicting the distribution of virulence genes among all isolates, where red indicates presence and green indicates absence. Virulence genes are shown in columns and bacterial isolates in rows.

The identified virulence genes are associated with various functions, including adherence, motility, effector delivery systems, nutritional/metabolic factors, immune modulation, invasion, and toxins. Among these, the genes related to the type III bacterial secretion system and siderophore uptake system were the most abundant, with 69 and 26 genes identified, respectively (Table 5).

**Table 5:** Summary of virulence genes.

Type of virulence factor	Virulence factor	Virulence gene	Number of genes
Adherence	Fimbrial	*fimA, fimB, fimC, fimD, fimE, fimF, fimG, fimH, fimI, yagV/ecpE, yagW/ecpD, yagX/ecpC, yagY/ecpB, yagZ/ecpA, paa, papB, papI, papX, sfaX, cfaA, cfaB, cfaC, cfaD/cfaE*	23
	Curli fibers	*cgsD, cgsE, cgsF, cgsG, csgA, csgB, csgC*	7
Motility	Flagellum	*fliG*	1
Effector delivery system	Type II secretion system	*gspC, gspD, gspE, gspF, gspG, gspH, gspI, gspJ, gspK, gspL, gspM*	11
	Type III secretion system	*escC, escD, escE, escF, escG, escI, escJ, escK, escL, escN, escO, escP, escR, escS, escT, escU, escV, espA, espD, espF, espG, espH, espK, espL1, espL2, espL4, espM1, espM2, espN, espO1-1, espO1-2, espP, espR1, espR4, espW, espX1, espX4, espX5, espX7/nleL, espY1, nleA/espI, nleB1, nleB2, nleC, nleE, nleF, nleG-1, nleG-3, nleG2-3, nleG2-4, nleG6-1, nleG7, nleG8-2, nleH1, nleH2, sepD, sepL, sepQ/escQ, map, glrA, glrR, lifA/efa1, cesAB, cesD, cesD2, cesF, cesL, cesT, toxB*	69
	Temperature sensitive hemagglutinin	*tsh*	1
	Non-fimbrial adhesin	*eae*	1
Nutritional/ Metabolic factor	Siderophore uptake system	*entA, entB, entC, entD, entE, entF, entS, fyuA/psn, iroB, iroC, iroD, iroE, iroN, irp1, irp2, fepA, fepB, fepC, fepD, fepE, fepG, iucA, iucB, iucC, iucD, iutA*	26
Immune modulation	Capsule	*gndA*	1
	TIR domain containing protein	*TcpC*	1
Invasion	AslA	*aslA*	1
	Invasion of brain endothelial cells	*ibeB, ibeC*	2
	K1 capsule	*kpsC, kpsD, kpsE, kpsF, kpsM, kpsS, kpsU*	7
	Outer membrane protein A	*ompA*	1
	TraJ	*AAA92657*	1
Toxin	Exotoxin	*hlyA, hlyB, hlyC, hlyD, stx2A, stx2B, cnf1, east1*	8

*E. coli* invades and colonizes extraintestinal tissues through its specific virulence factors, thereby establishing infections. Investigating the genes encoding these virulence factors in bacterial strains is crucial for determining their pathogenicity and elucidating the pathogenesis of infections. In this study, high-prevalence genes, particularly those from the *csg* (Curli fimbriae) and *fim* (Type I fimbriae) families, were widely detected. Curli fimbriae (encoded by *csgA*, *csgB*, *csgC*, *csgD*, *csgE*, *csgF*, and *csgG*) facilitate bacterial adhesion to yak host cells or biofilm formation, whereas Type I fimbriae (*fimA*, *fimB*, *fimC*, *fimD*, *fimE*, *fimF*, *fimG*, *fimH*, and *fimI*) enhance bacterial attachment to both biotic and abiotic surfaces, promoting biofilm formation and significantly increasing bacterial antibiotic resistance and environmental adaptability (36).

Flagella play a critical role in bacterial motility, chemotaxis, and host cell infection. During swarming motility, flagella assist bacterial adhesion to surfaces, facilitating initial biofilm formation and enhancing pathogenicity (37). In the yak intestinal tract, *E. coli* may utilize flagella-mediated movement to locate optimal colonization sites, followed by adhesion to intestinal epithelial cells and biofilm formation, thereby evading host immune clearance and sustaining diarrheal pathogenesis.

Virulence gene analysis revealed that the studied strains extensively carried genes associated with Type II (T2SS) and Type III secretion systems (T3SS). T2SS, widely present in Gram-negative bacteria, secretes substrate proteins that promote host cell adhesion, intoxication, and immune suppression, contributing to diverse diseases (38). T3SS, a multiprotein complex, translocates virulence effectors directly into host cells to disrupt cellular functions and mediate pathogenicity (39). These secretion systems likely facilitate *E. coli* colonization and pathogenesis in the yak intestine by delivering virulence factors that interfere with host cell physiology, ultimately inducing diarrhea.

Although toxins such as hemolysins (*hlyA*, *hlyB*, *hlyC*, *hlyD*) were detected at low prevalence (<35%), they may still significantly influence pathogenicity. Hemolysins lyse mammalian erythrocytes by forming pores in target cell membranes, leading to cell death. Consequently, these toxins may directly damage yak intestinal epithelial cells, compromise barrier function, and trigger inflammatory responses and diarrhea.

Given the high resistance to tetracyclines and the frequent detection of *tet*(A) and *tet*(B) (40), routine or empirical herd-level use of tetracyclines should be reduced. Ciprofloxacin and cefotaxime should not be used as routine first-line treatments because of the observed resistance and the public-health importance of fluoroquinolones and third-generation cephalosporins. Their use should be restricted to severe bacterial infections and guided by bacterial culture and antimicrobial susceptibility testing under veterinary supervision. Antimicrobial replacement should not involve automatic substitution within the same drug class. Instead, the narrowest effective antimicrobial legally approved for food-producing animals should be selected according to isolate susceptibility, farm-specific antibiograms, clinical indications, withdrawal periods, and national regulations. Because *aph*(6)-Id, *aph*(3″)-Ib, *sul*1, and *sul*2 were also frequently detected, aminoglycosides and sulfonamides should not be regarded as universal empirical alternatives.

Non-antimicrobial measures should include early isolation of diarrheic animals, appropriate supportive treatment, prompt removal of feces and contaminated bedding, mechanical cleaning before disinfection, correct disinfectant concentration and contact time, and complete drying of cleaned surfaces. Drinking troughs, pipelines, feeding equipment, and sick-animal areas should be regularly cleaned, with dedicated tools and improved manure, runoff, and water-quality management. Because 40% of isolates were strong biofilm producers, physical removal and prevention of biofilms on farm surfaces and water systems should be prioritized (41).

We also clarified that the high prevalence of *bla*_EC-18_ may partly reflect chromosomal AmpC variation and phylogenetic background rather than *β*-lactam exposure alone (42). Therefore, antimicrobial-use records, gene-expression studies, and genomic-context analyses are required for further interpretation.

### 3.4 Replicon analysis

Through comparison with the PlasmidFinder database, a total of 23 distinct plasmid replicons were identified. The overall carriage of plasmid replicons was relatively low, with most isolates carrying only one replicon. Notably, 7 strains lacked plasmid replicons, while 2 strains carried 7 replicons each (Figure 4). Compared with the plasmid replicon profiles of *E. coli* from poultry and other food-producing animals in China (43), the overall replicon carriage rate in yak-derived isolates was relatively lower, but the predominance of IncFIB replicons was consistently observed across different animal sources. The most prevalent replicon was IncFIB(AP001918)_1, detected in 55.00% (33/60) of isolates, followed by IFcFIA_1 with a carriage rate of 16.67% (10/60).

**Figure 4:**
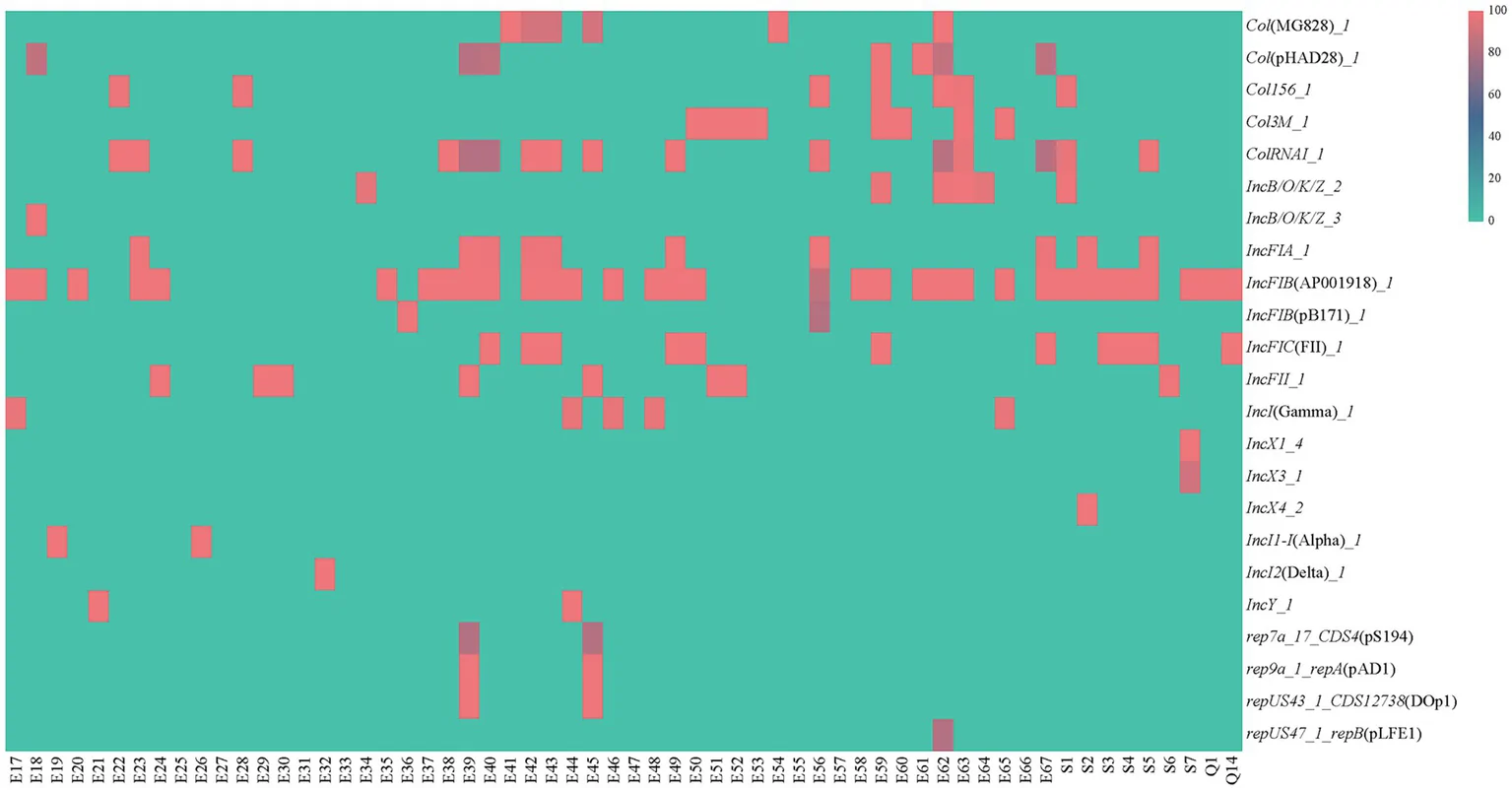
Heatmap of plasmid replicon types. A heatmap illustrating the prevalence of plasmid replicons across all isolates, with red denoting presence and green denoting absence. Bacterial isolates are displayed in columns and plasmid replicons in rows.

### 3.5 Phylogenetic and core-genome analysis

#### 3.5.1 Phylogenetic and phenotypic characteristics

In this study, the yak-derived *E. coli* isolates were predominantly classified as phylogroup B1 (81.67%, 49/60) (44), followed by phylogroup A (18.33%, 11/60). A total of 34 sequence types (STs) were identified, with 6 strains remaining untyped. The most prevalent ST was ST2522 (8.33%, 5/60), followed by ST56 and ST10 (5% each, 3/60). Twelve STs (ST2166, ST2035, ST1665, ST1642, ST1101, ST446, ST101, ST2179, ST6178, ST58, ST43, ST40) were detected in 2 strains each (3.33%), while 19 STs were found in single isolates (Figure 5B). Among these, 7 STs belonged to CC155 and 5 STs belonged to CC10. Seventeen serotypes were identified, with O25 and O107 being the most prevalent (16.67% each, 10/60), followed by O26 and O55 (8.33% each, 5/60). Biofilm formation analysis (Figure 5A) showed that 40% (24/60) of strains exhibited strong biofilm-forming capacity, 51.67% (31/60) showed moderate capacity, 6.67% (4/60) were weak formers, and 1.67% (1/60) were non-biofilm formers.

**Figure 5:**
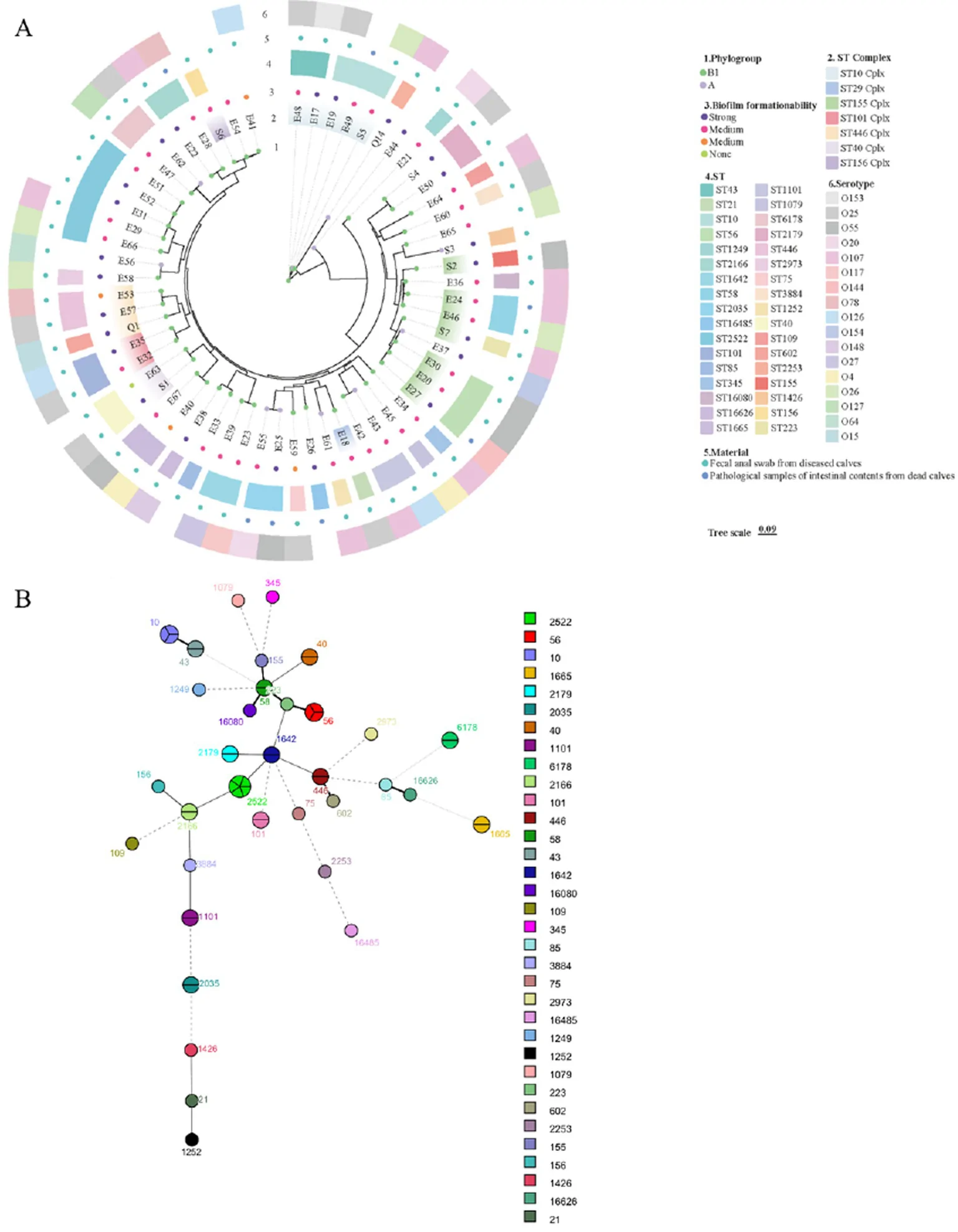
Phylogenetic analysis. **(A)** Circular tree showing the phylogenetic relationship of the 60 *E. coli* isolates. The tree was generated based on the core genome SNPs. **(B)** Analysis of the minimum spanning tree of the 60 sequenced *E. coli* isolates based on the sequence type. Each node represents a unique sequence type ST, with the circle size proportional to the number of isolates sharing the same ST.

Phylogroup B1, which accounted for 81.67% of our isolates, is frequently associated with the intestinal microbiota of cattle and other ruminants and is generally regarded as a host-generalist lineage. Although many B1 strains are commensal, this phylogroup can include diarrheagenic or opportunistic isolates carrying antimicrobial-resistance and virulence determinants. Its predominance may therefore reflect adaptation to the ruminant intestinal niche rather than uniformly high pathogenicity.

Among the major sequence types, ST10 is a widely distributed, broad-host-range lineage recovered from humans, livestock, food, and environmental sources. Its frequent association with acquired resistance genes and mobile genetic elements highlights its importance at the human-animal-environment interface. ST56 has also been identified in cattle, calves, pigs, sheep, and other animal-associated collections, including diarrheic-animal isolates, suggesting adaptation to livestock hosts; however, its specific contribution to yak diarrhea requires functional confirmation. ST2522 remains less extensively characterized but has been reported from livestock, food, and environmental sources. Therefore, its relatively high frequency in our collection suggests possible regional enrichment rather than yak specificity.

Integrating phylogroup, MLST, serotype, resistance, and biofilm data, the Qinghai yak-derived *E. coli* population appears to comprise a diverse B1-dominated assemblage containing broadly distributed lineages, such as ST10 and ST56, together with a potentially locally enriched ST2522 lineage. However, because ST2522, ST56, and ST10 were represented by only five, three, and three isolates, respectively, they should be described as dominant detected lineages rather than confirmed epidemic clones. We have accordingly revised the text and emphasized that cgMLST or whole-genome SNP analysis, combined with temporal, geographic, environmental, and human sampling, is required to confirm clonal transmission and One Health dissemination.

#### 3.5.2 Biofilm formation capacity and strain characteristics

Among the strong biofilm-forming *E. coli* isolates (45), phylogroup B1 predominated (70.83%, 17/24), followed by phylogroup A (29.17%, 7/24). Eleven serotypes were identified in this group, with O25 being the most prevalent (20.83%, 5/24). The most common sequence types were ST2522 and ST2179 (4.17% each, 2/24). For moderate biofilm formers, phylogroup B1 again showed dominance (87.10%, 27/31), while phylogroup A accounted for 12.90% (4/31). Ten serotypes were detected, with O107 being predominant (22.58%, 7/31), followed by O25 and O26 (12.90% each, 4/31). ST2522 was the most prevalent sequence type (9.68%, 3/31). The weak biofilm-forming group comprised 4 isolates, all belonging to phylogroup B1. These exhibited four serotypes (O4, O78, O126, and O25). One strain was untyped, while the remaining three showed ST1665, ST446, and ST75. Only one non-biofilm forming strain was identified, which was phylogroup B1, had undetermined serotype, and belonged to ST40. Given the high prevalence of biofilm formers among the isolates, alternative strategies to combat biofilms on farms and in food production deserve attention; these include the use of bacteriophages as antibiofilm agents (46), immunomodulatory substances such as interleukin-2 components that may affect biofilm formation in uropathogenic *E. coli* (47), and nanoparticle-based approaches, including Fe-doped ZnO nanoparticles with antibacterial efficacy against multidrug-resistant *E. coli* (48).

#### 3.5.3 Correlation between biofilm formation capacity and antibiotic resistance genes

Among the strong biofilm-forming *E. coli* isolates (n = 24), we detected 50 resistance genes belonging to 9 classes. The most prevalent was the *β*-lactam resistance gene *bla*_EC-18_ (79.17%, 19/24), followed by sulfonamide resistance gene *sul1* (41.67%, 10/24), tetracycline resistance gene *tet*(A) (37.50%, 9/24), and aminoglycoside resistance gene *aph*(6)-*Id* and sulfonamide gene *sul2* (33.33% each, 8/24). Twenty resistance genes were each detected in only one isolate. The moderate biofilm-forming isolates (n = 31) carried 42 resistance genes from 10 classes, with *bla*_EC-18_ showing the highest prevalence (90.32%, 28/31), followed by aminoglycoside genes *aph*(6)-*Id* and *aph*(3″)-*Ib* (38.71%, 12/31), *sul2* (35.48%, 11/31), and *tet*(B) (29.03%, 9/31). Nineteen genes were unique to single isolates. Weak biofilm-formers (n = 4) harbored 10 resistance genes across 8 classes, with *bla*_EC-18_ remaining dominant (75%, 3/4) and quinolone resistance gene *qnrD1* (50.00%, 2/4); eight other genes each appeared in one isolate. The single non-biofilm forming isolate carried only two genes: *bla*_EC-18_ and *qnrD1*.

Despite substantial heterogeneity in animal health status, sample sources, antimicrobial panels, susceptibility criteria, and study periods, several common patterns were identified. Tetracycline resistance was consistently among the most prevalent phenotypes across livestock species and regions, indicating widespread and sustained selection pressure from this antimicrobial class. Resistance to fluoroquinolones and third-generation cephalosporins was generally more variable, suggesting that local treatment practices, production systems, and antimicrobial exposure strongly influence regional resistance profiles. Aminoglycoside- and sulfonamide-resistance rates also differed considerably among studies, further supporting the need for region-specific surveillance rather than uniform empirical treatment recommendations.

Regional comparisons showed that yak-derived isolates from Qinghai and Tibet generally exhibited higher ciprofloxacin resistance than those reported from Sichuan, whereas some Sichuan isolates showed greater resistance to trimethoprim–sulfamethoxazole or aminoglycosides. However, these differences should be interpreted cautiously because diarrheic and healthy animals were not consistently distinguished across studies. Serotype data were comparatively limited and heterogeneous; in our collection, O25 and O107 predominated, but broader multi-regional evidence is insufficient to confirm that these serotypes are universally dominant in Chinese livestock. Likewise, biofilm formation was common in the available studies, although differences in experimental methods limited direct quantitative comparison.

Overall, tetracycline resistance appears to be the most consistent cross-regional feature, whereas fluoroquinolone, cephalosporin, aminoglycoside, and sulfonamide resistance show stronger regional variation. These findings support reduced empirical tetracycline use, local antibiogram-guided therapy, and standardized national surveillance integrating resistance, serotype, biofilm phenotype, host species, and animal health status.

#### 3.5.4 Virulence factor distribution across biofilm-forming phenotypes

Strong biofilm-forming *E. coli* (49) (*n* = 24) carried 198 virulence factors, with 26 genes (*acrB*, *cgsG*, *csgA*, *csgC*, *entA-E*, *espL1*/*X4*/*X5*, *fdeC*, *fepC*/*D*/*G*, *fur*, *gndA*, *hcp1*/*tssD1*, *ibeB*/*C*, *ompA*, *phoP*, *pmrA*, *rpoS*, *tssA*) showing 100% prevalence (24/24). An additional 18 genes (*allB*, *cgsD*, *entF*/*S*, *espR1*, *fepA*/*B*/*E*, *fes*, *fimA*/*C*-*I*, *gspM*, *rcsB*, *yagZ*/*ecpA*) were detected in 95.83% (23/24) isolates. Notably, 34.85% (69/198) of virulence factors appeared in only one strain.

Moderate biofilm-formers (*n* = 31) harbored 237 virulence factors. Twenty-six genes (*acrB*, *cgsD-G*, *csgA-C*, *entD*, *espX4*, *fdeC*, *fepA-C*/*G*, *fes*, *fimA-C*/*E*/*F*, *fur*, *gndA*, *phoP*, *rpoS*, *yagV*/*ecpE*) were universal (100%, 31/31), while 23 genes (*allB*, *entA-C*/*E*/*F*/*S*, *espL1*/*R1*, *fepD*/*E*, *fimD*/*H*/*I*, *ibeB*/*C*, *ompA*, *pmrA*, *rcsB*, *yagW-Z*/*ecpA-D*, *ykgK*/*ecpR*) reached 96.77% (30/31) prevalence. 38.40% (91/237) were strain-specific.

Weak biofilm-formers (*n* = 4) contained 102 virulence factors, with 42 genes (*allB*, *cfaA*/*B*/*D*/*E*, *cgsD-G*, *csgA-C*, *entB*/*D*/*S*, *espR1*, *fdeC*, *fepB*, *fimA*/*B*/*E-I*, *gndA*, *gspE-M*, *ompA*, *phoP*, *pmrA*, *rcsB*, *rpoS*, *yagV*-*Z*/*ecpA*/*B*/*D*, *ykgK*/*ecpR*) present in all isolates (100%, 4/4). Thirty-four genes (*acrB*, *cfaC*, *entA*/*C*/*E*/*F*, *espL1*/*X1*/*X4*/*X5*, *fepA*/*C*-*E*/*G*, *fes*, *fha*, *fimC*/*D*/*G*, *fur*, *gspC*/*D*, *hcp1-2*/*tssD1-2*, *ibeB*/*C*, *tssA*/*B*/*F*/*G*/*J*/*L*, *vgrG*/*tssI*, *yagX*/*ecpC*) showed 75% (3/4) detection. Non-biofilm formers (*n* = 1) carried 139 virulence factors, though statistical significance was limited due to sample size.

Moreover, by analyzing the correlation between biofilm-forming capacity, ARGs and virulence genes, it was found that biofilm-forming capacity was not significantly correlated with either ARG diversity (Spearman’s *p* = 0.168, *p* = 0.200) or virulence gene carriage rate (Spearman’s *p* = 0.075, *p* = 0.571). In addition, no significant correlation was observed between ARG diversity and virulence gene carriage rate (Spearman’s *p* = 0.126, *p* = 0.336).

Curli fimbriae, mainly encoded by the *csg* operon, mediate adhesion to extracellular matrix proteins such as fibronectin and laminin and promote interbacterial aggregation. The universal detection of *csgA* and *csgC* suggests that most isolates possess the genetic potential to establish initial attachment to the yak intestinal mucosa, resist peristaltic clearance, and promote biofilm formation. Type I fimbriae, represented by the highly prevalent *fimA* gene, further support close attachment to mannosylated receptors on intestinal epithelial cells. This intimate contact may stabilize colonization and facilitate the local action of secretion-system effectors and toxins.

Hemolysin genes (*hlyA-hlyD*) were detected in fewer than 35% of isolates but may confer enhanced pathogenic potential. *HlyA* is a pore-forming cytotoxin that disrupts host-cell membranes and can damage intestinal epithelial cells, erythrocytes, and immune cells. Such injury may compromise epithelial-barrier integrity, increase intestinal permeability, induce local inflammation, and promote fluid and electrolyte leakage into the intestinal lumen, thereby contributing to diarrhea. Curli- and type I fimbriae may therefore primarily support adhesion, persistence, and biofilm development, whereas hemolysin may intensify tissue damage after colonization.

Taken together, the widespread carriage of adhesion genes and the presence of toxin genes in a subset of isolates indicate a potential stepwise pathogenic process involving mucosal attachment, persistent colonization, epithelial injury, and diarrheal symptoms. However, gene carriage alone does not demonstrate expression or causality. We have therefore used cautious language and emphasized the need for adhesion, cytotoxicity, hemolysis, gene-expression, and yak intestinal epithelial-cell assays to validate these proposed mechanisms.

## 4 Conclusion

This study reveals that *E. coli* from diarrheic yaks in Qinghai exhibit: (i) high multidrug resistance, particularly to tetracyclines (43.33%), ciprofloxacin (33.33%), and cefotaxime (33.33%), with *bla*_EC-18_ (85%) as the dominant resistance gene; (ii) abundant virulence potential, with universal carriage of adhesion genes (*csgA*, *fimA*) and detection of toxin genes (*hlyA*, *stx2A*); (iii) high biofilm-forming capacity (91.67%), with strong formers carrying greater ARG and virulence gene diversity; and (iv) predominance of phylogroup B1 (81.67%), ST2522, ST56/ST10, and serotypes O25/O107. However, several limitations should be acknowledged, (i) a small sample size from a single region; (ii) lack of whole-genome sequencing to confirm the genetic context of *bla*_EC-18_ and assess horizontal transfer risk; (iii) absence of farm-level antimicrobial usage data to directly link resistance to prescribing practices; and (iv) lack of functional assays to validate virulence gene expression and pathogenicity. To address these gaps, future studies should conduct large-scale, multi-regional surveillance with standardized protocols, employ whole-genome sequencing for high-resolution phylogenetic analysis, integrate antimicrobial usage data to inform evidence-based stewardship, and implement One Health surveillance encompassing yaks, farm environments, and human populations. In addition, biofilm-targeted control strategies, including optimized cleaning and disinfection protocols, warrant further investigation to mitigate the risks posed by antimicrobial-resistant and pathogenic *E. coli* in the Qinghai-Tibet Plateau.

In summary, this work highlights that yak-derived *E. coli* serve as a reservoir of resistance and virulence determinants, calling for evidence-based interventions and integrated One Health monitoring to safeguard both animal and human health in the Qinghai-Tibet Plateau.

## Data Availability

The data presented in the study are deposited in the NCBI SRA repository, accession number PRJNA1502479.

## Footnotes

1
http://www.genomicepidemiology.org/
